# Body iron and lead status in early childhood and its effects on development and cognition: a longitudinal study from urban Vellore

**DOI:** 10.1017/S1368980019004622

**Published:** 2020-08

**Authors:** Beena Koshy, Manikandan Srinivasan, Susan Mary Zachariah, Arun S Karthikeyan, Reeba Roshan, Anuradha Bose, Venkata Raghava Mohan, Sushil John, Karthikeyan Ramanujam, Jayaprakash Muliyil, Gagandeep Kang

**Affiliations:** 1 Developmental Paediatrics Unit, Christian Medical College, Vellore, Tamil Nadu 632004, India; 2 Wellcome Research Unit, Christian Medical College, Vellore, Tamil Nadu 632004, India; 3 Community Health, Christian Medical College, Vellore, Tamil Nadu 632004, India; 4 Low Cost Effective Care Unit, Christian Medical College, Vellore, Tamil Nadu 632004, India

**Keywords:** Early childhood, Child nutrition, Child development, Cognition, Iron deficiency, Lead toxicity

## Abstract

**Objective:**

Early childhood factors can have persisting effects on development and cognition in children. We propose to explore the trends of Fe deficiency and Pb toxicity in early childhood and their association with child development at 2 years of age and cognition at 5 years.

**Design:**

Longitudinal birth cohort study.

**Setting:**

Urban slum, Vellore, India.

**Participants:**

Children enrolled at birth were followed up regularly in the first 2 years with developmental and cognitive assessments at 2 and 5 years of age, respectively.

**Results:**

The birth cohort enrolled 251 children with 228 children followed up at 2 years and 212 at 5 years of age. Fe deficiency (ID) was highest at 15 months of age and improved subsequently at 24 months. Blood Pb levels (BLL) remained high at all age groups with an increasing trend with age; 97 % at 36 months having high BLL. Persistent high mean BLL at 15 and 24 months had negative association with both cognition and expressive language raw scores of 24 months, while high mean BLL at 15, 24 and 36 months had no significant association with any of the domains of cognition at 5 years of age. Early childhood cumulative body Fe status at 7, 15 and 24 months did not show any association with child development at 2 years, but was associated with verbal, performance and processing speed components of cognition at 5 years.

**Conclusions:**

Optimising body Fe status and limiting Pb exposure in early childhood can augment child development and school entry cognition.

It is now recognised that the ‘first 1000 days of life’, from conception to the second birthday, are critical for brain development. This has resulted in an extensive exploration of early childhood factors to understand their potential impact on child development and sequentially human potential^(1)^. There are many genetic, environmental and epigenetic factors that affect child’s development, and it is challenging to identify a single factor, the deficiency of which is common, easy to detect and treat, and amenable for community-level interventions.

Micronutrient deficiencies such as that of Fe and I, especially in early infancy, can have long-term effects on cognition and behaviour, probably due to their impact on the rapidly developing brain during the ‘first 1000 days of life’^(2,3)^. Fe deficiency (ID) is considered as the most common nutritional deficiency among all age groups and is prevalent across all social strata^(2,4)^. Fe is vital, not just for the synthesis of Hb but also for the non-haematological effects on mitochondrial and neuroendocrine function^(4)^. ID therefore affects cognition and behaviour in children, and these changes can be long term. Although there are studies evaluating anaemia and Fe status and their effects on cognition, there is a dearth of evidence, of the long-term effect of ID in early childhood, especially from low–middle income countries.

In addition to micronutrient deficiency, Pb exposure in early childhood can adversely affect child development including cognition. Even low levels of Pb exposure during the early childhood brain development stage can have lasting effects on cognition and behaviour^(5)^. With increasing recognition of the adverse effects of high Pb levels on young brains, there has been a revision of the cut-off for elevated blood Pb levels (BLL) from 0·48 µmol/l (10 µg/dl) to 0·24 µmol/l (5 µg/dl) by the American Academy of Pediatrics^(6,7)^. Background deficiency of micronutrients particularly that of Fe can modify the impact of exposure to high levels of Pb through pica and competitive inhibition of intestinal absorption^(8,9)^. A previous study from the same birth cohort had reported high BLL at 15 and 24 months of age in children^(10)^. The objective of the current study was to evaluate the trends of both Fe and Pb status in early childhood and the association of cumulative early childhood Fe and Pb status on development at 2 years of age and cognition at 5 years of age.

## Materials and methods

### Study population and methodology

The study population was a birth cohort followed up for the Etiology, Risk Factors and Interactions of Enteric Infections and Malnutrition and the Consequences for Child Health and Development (MAL-ED) Network, a multinational, longitudinal prospective cohort study conducted in eight countries across the world^(11)^. The study site in India was eight adjacent urban slum dwelling areas, located in Old Town, Salavanpet and neighbouring areas in Vellore, South India, with a population density of 42 000/km^2(12)^, compared with 6200/km^2^ in Rio de Janeiro and 32 000/km^2^ in Harlem, NYC. A population of 12 000 was covered for the current study. All pregnant women in the study site were identified by door-to-door survey and invited to participate in the study immediately after the delivery, by consecutive sampling. The enumeration process carried out prior to recruitment provided the probable number of births in the study area based on recent births and the number of pregnant mothers. Using this information, it was planned to recruit more than 200 children over the 2-year period, the target number for each site as per the MAL-ED study protocol (for details, refer to John *etal*.^(12)^). Mothers were screened based on the following exclusion criteria – having pre-existing plans for migration during the study period, having another child from the same family being enrolled in the MAL-ED study, multiple pregnancies, having a neonate with medical complications and unavailability of mothers to provide informed consent. Babies of mothers who consented were enrolled, and their demographic characteristics along with anthropometry measurements were recorded. Babies were enrolled at a mean (range) age of 10·08 (0–16) d. Trained fieldworkers visited their home twice a week for active disease surveillance for the first 2 years of life. The initial birth cohort recruitment was conducted between March 2010 and February 2012 and recruited 251 children, and baseline demographic data of the birth cohort were comparable to that of the study area^(12)^. The cohort was further followed up at 36 and 60 months of age.

Blood samples were collected at 7, 15, 24, 36 and 60 months. Samples were tested for Hb (at 7, 15, 24, 36 and 60 months); Fe and ferritin assays (at 7, 15 and 24 months) and for BLL (at 15, 24 and 36 months). All children underwent a developmental assessment at 7, 15, 24 and 36 months of age using Bayley Scales of Infant and Toddler Development-3rd edition (BSID-III) and cognitive assessment at 5 years of age, using Wechsler Preschool Primary Scales of Intelligence – third edition (WPPSI-III). Child development is defined as the ability of the child in domains of motor, language, cognition, play, social and adaptive skills^(13)^. Cognition or intelligence is defined as a combination of multiple abilities in a child^(14)^. The original study and further follow-up studies were approved by the institutional review board. The current study was conducted according to the guidelines laid down in the Declaration of Helsinki, and all procedures involving human subjects namely growth monitoring, cognition assessments and blood collection were approved by the institutional review board, Christian Medical College, Vellore, India. Written informed consent was obtained from the parents/caregivers of all the subjects.

## Study measures

### Blood collection

Venous blood was collected from children to a maximum quantity of 5 ml at each visit. Hb was analysed using a point-of-care screening tool, Hemocue^®^ Hb 301 kit (azide-methemoglobin method), at the study clinic immediately after blood collection using a drop of blood. One millilitre of the collected venous blood was used for BLL analysis and preserved with K2 EDTA, and the remaining blood was stored in the biochemistry tube for analysis of serum ferritin and transferrin levels. The samples were refrigerated and transported using cold packs to the research laboratory within 2 h of blood collection for further analysis. Serum ferritin, transferrin receptors and Pb levels were tested in the Biochemistry department, Christian Medical College, Vellore. Chemiluminescence immunoassay was done for serum ferritin analysis using Siemens Advia Centaur (Model Siemens Advia Centaur Xp Chemiluminescence immune assay) with 0·5 μg (11·24 pmol) as limit of detection. For estimating transferrin levels, Immunoturbidimetry was performed using Roche Coba C system (model Roche Cobas C 702 Immunoturbidimetry assay) with 10 μg (0·13 µmol) as limit of detection. Using the Graphite Furnace Atomic Absorption Spectroscopy method in M-series Atomic Absorption Spectro-photometer (model Thermo Scientific X series 2.ICP-MS), BLL were estimated with 0·5 μg (0·024 µmol) as limit of detection.

### Bayley Scales of Infant and Toddler Development-III

The BSID-III assesses development in the domains of gross motor, fine motor, expressive language, receptive language and cognition between 1 and 42 months of age^(13)^. The measure was culturally adapted, translated and piloted prior to administration. A single trained psychologist conducted the testing in a standardised child friendly setting in the community clinic for each age group. Around 5–6 % of the tests were video-recorded and centrally reviewed for quality control purposes^(15)^. For the purpose of current analysis, the 24-month assessment was used, as 2 years denote the completion of infancy and the ‘1000-day window’ period^(1)^. Receptive and expressive language and cognition domain raw scores were derived for each child from the 24-month assessment. Receptive and expressive language skills measure receptive and expressive abilities of children in primary language and communication areas, while cognition measures sensorimotor development, exploration and manipulation, object relatedness, concept formation and memory^(13)^.

### Wechsler Preschool and Primary Scales of Intelligence-third edition

The WPPSI-III can be administered to children aged between 2 years 6 months and 7 years 3 months and assesses cognition in the domains of verbal, performance and processing speed. This measure was adapted centrally for cultural appropriateness and was translated and piloted in local settings before being administered by a single trained psychologist. Verbal, performance and processing speed raw scores were obtained after administration for each child. Ten percentage of these assessments were recorded and reviewed centrally for quality control.

Maternal intelligence was assessed at 6–8 months of child age by Raven’s progressive matrices^(16)^. This time of assessment was chosen in the protocol for mothers’ convenience as they would be able to leave their babies with another caregiver around this time, to complete cognitive assessment in the study clinic. To assess the socio-economic status (SES), a socio-economic index was created using Water and sanitation, Assets, Maternal education and household Income (WAMI) and SES of the families was assessed at recruitment and six-monthly intervals as per the study protocol^(17)^. The WAMI index was validated against prevalence of stunting in all the eight countries involved in the MAL-ED cohort study^(17)^. The broader aim of the main study was to assess the predictors of early child development, amongst which SES influences child development through both biological and physical home environment^(11,18)^.

As serum ferritin levels can be influenced by inflammation, total body Fe levels were calculated from ferritin (F) and transferrin (R) levels as below^(19)^:

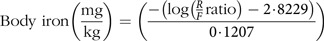




Positive values indicated Fe surplus in stores, while negative values indicated deficiency of Fe in tissues.

## Data entry and analysis

The Data Coordinating Center developed a double data entry database application. Forms were checked for completeness and accuracy of the data by data supervisors before being entered into the application. Discrepancies between the first and second entry were cleared by data supervisors after rechecking forms, and this process of cross validation was done for quality control of the study. The data was analysed using STATA 13 (StataCorp). Missing data values were limited as this was a prospective study with rigorous quality checks. If a child did not have information at one time point, that child was excluded from corresponding analysis and no missing value corrections were applied.

Mean and sd were used to summarise Hb, Pb, ferritin and transferrin levels measured at each time point. A birth weight <2·5 kg was considered as low birth weight category. We reported children who were anaemic (Hb < 11 g/dl)^(20)^, Fe deficient (blood Fe < 0 mg/kg) and with elevated BLL (>0·24 μmol/l) using proportions. Characteristics of the study children such as sex and SES at 2nd and 5th year of follow-up were compared with baseline data using ‘Chi-square for trends’. Mean values of Hb across the time points (7, 15, 24, 36 and 60 months) were compared using repeated-measures ANOVA. Bonferroni test was used for *post hoc* analysis, to compare the Hb levels between any of the two time points measured. Estimates of median serum ferritin, transferrin and BLL across each time point were compared using Friedman’s test, and *post hoc* analysis between any of the two points was done using Wilcoxon Signed-rank test. Proportions of children with anaemia, ID and elevated Pb levels across each time point were compared using ‘Chi-square for trends’. The proportion with ID in anaemic children was evaluated to understand causes of anaemia in each age group setting. The outcome variables namely child development (BSID-III) measured at 2 years were expressed as raw scores of cognition, expressive language and receptive language domains, and cognition (WPPSI-III) measured at 5 years was expressed as raw scores of verbal, performance and processing speed domains. Median (IQR – interquartile range) was used to summarise the scores under each domain of the outcome variables. Cronbach’s alpha was used to assess the internal consistency within items under each domain of outcome measures to evaluate if the responses provided by the subject remained consistent. Since Fe and Pb levels were analysed at different time points, a summary statistic approach was used and the repeatedly measured information was condensed to a single number per subject^(21,22)^. Mean body Fe was derived by averaging the body Fe levels at 7, 15 and 24 months, and this measure was considered for analysis at both 2 and 5 years. Mean BLL was derived by averaging the BLL at 15 and 24 months for analysis at 2 years, and those at 15, 24 and 36 months for analysis at 5 years.

Linear regression modelling was done to find the predictors of the outcome variables – BSID-III and WPPSI-III scores using separate models. The predictors that were included in the model were – sex of the child, maternal intelligence raw scores, SES, mean body Fe levels and mean BLL. In the bivariate analysis, unadjusted beta coefficient along with 95 % CI was reported for each predictor. A multivariable linear regression was performed using the same set of predictors listed above. Following assumptions of linear regression were tested – normality of the outcome variable using Shapiro–Wilk test and multicollinearity of predictors using correlation matrix were tested. Only those independent variables with the correlation coefficient (*r* value) of <0·5 in the multicollinearity analysis were retained in the model. The standardised beta coefficient along with 95 % CI of the predictor variables was noted. In the post-estimation analysis, homoscedasticity of the residuals was assessed using Breusch–Pagan test. The level of significance for the test of association was considered at *P* < 0·05.

## Results

**Fig. 1:**
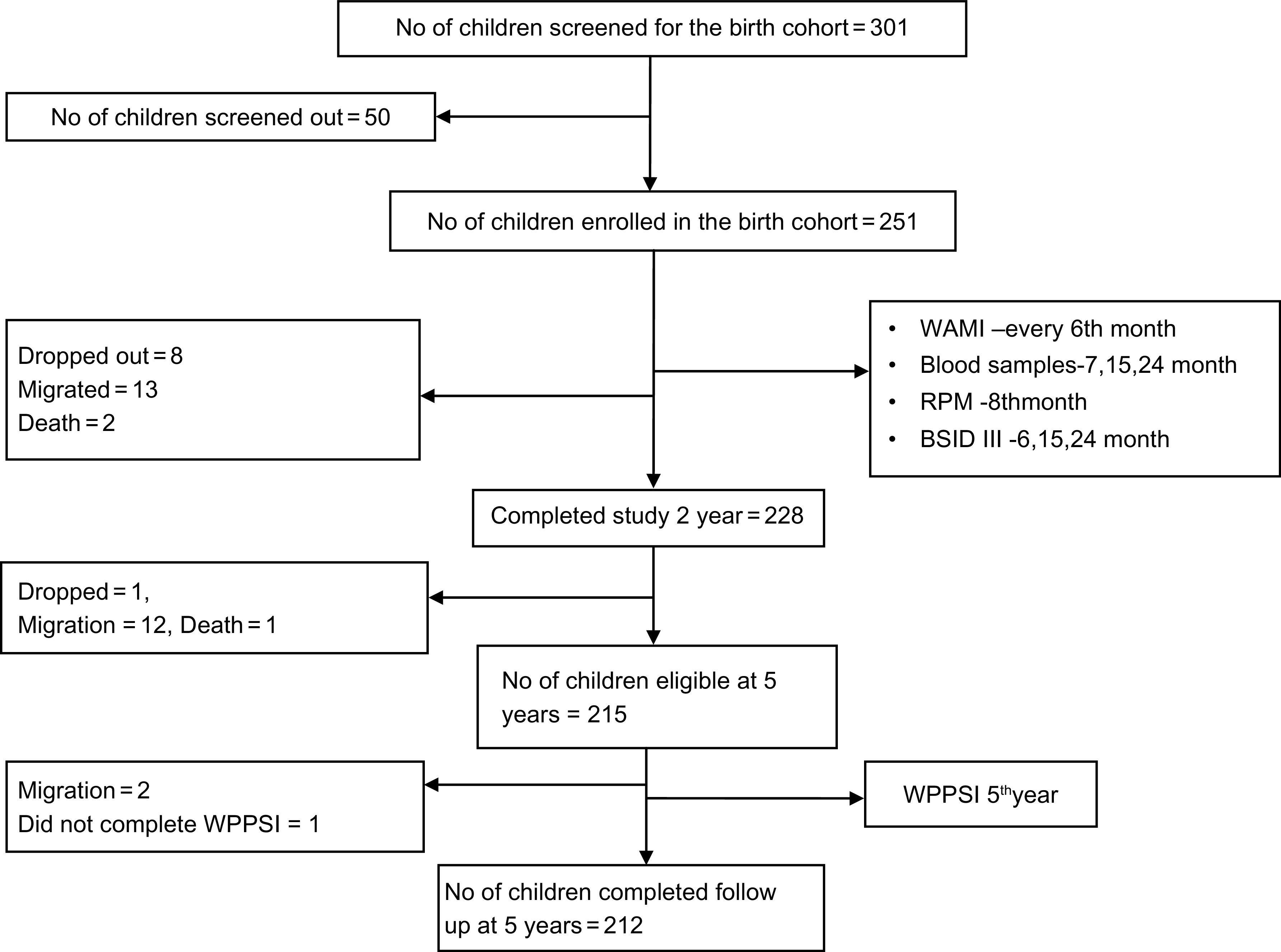
Flow chart depicting the follow-up of the birth cohort. WAMI, Water and sanitation, Assets, Maternal education and Income score for socio-economic status; RPM, Raven’s progressive matrices; BSID III, Bayley Scales of Infant and Toddler Development III; WPPSI III, Wechsler Preschool Primary Scales of Intelligence

After visiting 301 pregnant mothers, 251 children were enrolled at birth after parental consent, as ten mothers refused to participate and forty mothers did not fulfil the screening criteria (screen failures). Reasons for screen failures included pre-existing plans for migration during the study period (*n* 5), another child of the same family being part of the MAL-ED study (*n* 8), multiple pregnancy (*n* 1), presence of pre-existing morbidities in children (*n* 7) and mothers not available to give informed consent (*n* 9) and a combination of two or more of the above reasons (*n* 10). On follow-up, 228 children participated at 2 years of age and 212 children at 5 years of age (Fig. 1) and there was no significant difference in the socio-demographic characteristics between participants at baseline and at 2 or 5 years of age follow-ups (Table 1). Loss to follow-up was mainly due to migration of the families (Fig. 1).

**Table 1:** Characteristics of study children at enrolment, 2 and 5 years of age

Characteristics	Enrolment (*N* 251)	At 2 years (*N* 228)	At 5 years (*N* 212)	*P* *
Sex				
Male				
*n*	113	105	98	0·960
%	45·0	46·0	46·2	
Female				
*n*	138	123	114	
%	55·0	54·0	53·8	
SES (WAMI)				
Low				
*n*	71†	71	65	0·955
<33rd centile	30·2	31·1	30·7	
High				
*n*	164†	157	147	
≥33rd centile	69·8	68·9	69·3	
Maternal IQ – raw score				
Median		46·5	45	–
Range		8–68	8–68	–

SES, socio-economic status; WAMI, Water and sanitation, Assets, Maternal education and Income score; IQ, intelligence quotient.

* Using *χ* ^2^ for trends.

† Baseline SES data were available only for 235 children.

The birth cohort had a mean (range) family size of 5·7 (3–13) and the mean (range) educational year of the head of the family was 6·8 (0–18)^(12)^. The mean BMI of mothers was 22 (sd 3·9) kg/m^2^ with 20·3 and 21·1 % of mothers being underweight and overweight/obese, respectively. The cohort participants were predominantly girls (56 %), and the mean birth weight was 2·8 (sd 0·44) kg. Seventeen percentage of babies were low birth weight and weighed <2·5 kg at birth. The mean exclusive breast-feeding duration was 76·9 (sd 43·8) d.

There was a reduction in Hb levels between 7 and 15 months; however, this reduction was not significant (*P* = 1·000) (Table 2). Subsequently, there was a significant increase in the Hb levels at 15, 24 and 36 months (15 *v*. 24 months, *P* = 0·012; 24 *v*. 36 months, *P* = 0·0001). Although ferritin levels significantly dropped between 7 and 24 months (*P* < 0·0001), there was no change at 36 months (15 *v*. 36 months, *P* = 0·3745). There was a significant increase in mean BLL across the three time points (15 *v*. 24 months, *P* < 0·0001; 24 *v*. 36 months, *P* = 0·0001) (Table 2).

**Table 2:** Trend of Hb, ferritin, transferrin and lead levels at different time points of the cohort follow-up

Indicator	7 months			15 months			24 months			36 months			60 months			*P*
	*N*	Mean	sd	*N*	Mean	sd	*N*	Mean	sd	*N*	Mean	sd	*N*	Mean	sd	
Hb (gm/dl)	233	10·8	1·2	228	10·6	1·3	225	10·9	1·4	112	11·5	1·2	159	11·8	0·9	<0·0001
Ferritin (pmol/l)	230	91·2	123·4	221	33·9	45·6	222	41·6	60·4	NA	NA		NA	NA		<0·0001
Transferrin (µmol/l)	218	16·8	11·2	226	27·1	20·7	225	27·6	20·7	NA	NA		NA	NA		0·001
Pb (µmol/l)	NA	NA		228	0·5	0·3	223	0·6	0·4	113	0·6	0·3	NA	NA		0·002

*N*, total number of children whose blood samples were available for analysis at respective time points; NA, not available.

**Fig. 2:**
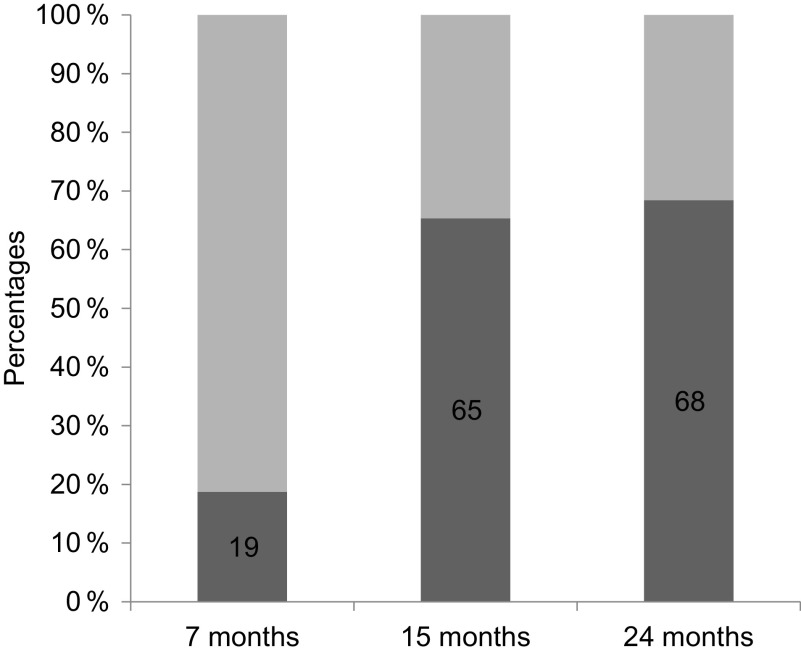
Proportion of iron deficiency in anaemic children at 7, 15 and 24 months of age. 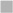, Anaemia due to caused other than iron deficiency; 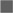, iron deficiency anaemia

More than half of the children were anaemic at 7 and 15 months of age, with subsequent improvements in Hb levels at 24, 36 and 60 months. Almost half of the study group (46·1 %) had low Fe status at 15 months of age (Table 3). Across the time points, there was an increase in proportion of anaemia attributable to ID from 19 % at 7 months to 68 % at 24 months (Fig. 2).

**Table 3:** Percentage of children with anaemia (Hb < 11 gm/dl), low body iron stores and high blood lead level (>0·24 µmol/l)

Indicator	7 months		15 months		24 months		36 months		60 months		
Hb status	*N*	%	*N*	%	*N*	%	*N*	%	*N*	%	*P*
Children with anaemia	120	51·5	129	56·6	96	42·7	35	31·3	24	15·1	<0·0001
Children without anaemia	113	48·5	99	43·4	129	57·3	77	68·7	135	84·9	
Body Fe status											
Children with Fe deficiency	24	11·1	102	46·1	96	42·9	NA		NA		<0·0001
Children without Fe deficiency	192	88·9	119	53·9	128	57·1	NA		NA		
Blood level status											
Children with elevated blood Pb level	NA		228	89·9	214	95·9	110	97·3	NA		0·004
Children without elevated blood Pb level	NA		23	10·1	9	4·1	3	2·7	NA		

*N*, the total number of children whose blood samples were available for analysis at respective time points; NA, not available.

The Cronbach’s alpha for internal consistency was 0·927, 0·712 and 0·727 for expressive language, receptive language and cognition domains of BSID-III at 2 years of age, respectively, and 0·789, 0·705 and 0·802 for verbal, performance and processing speed domains of WPPSI-III at 5 years of age, respectively. Median raw scores for expressive language, receptive language and cognition were 27 (IQR 16–33), 29 (IQR 15–36) and 59 (IQR 47–69), respectively, at 2 years of age, while the median raw scores at 5 years of age for verbal, performance and processing speed domains were 39 (IQR 13–69), 48 (IQR 22–75) and 35 (IQR 1–86), respectively (Table 4).

**Table 4:** Outcome raw scores of development and cognition using Bayley Scales of Infant and Toddler Development III (BSID III) and Wechsler Preschool Primary Scales of Intelligence IV (WPPSI IV) measured at 2 and 5 years, respectively

	Mean	sd	Median	IQR
Development scores at 2 years (*N* 227*)				
Cognition	59·5	3·4	59	57–62
Expressive language	27·8	4·7	29	25–31
Receptive language	26·9	2·5	27	25–29
Cognition scores at 5 years (*N* 212)				
Verbal	37·9	9·2	39	32–44
Performance	48·2	9·8	48	42–54
Processing speed	35·8	18·4	35	22–50

IQR, interquartile range.

* Of the 228 children who completed 2 years of follow-up, one subject did not have complete developmental raw scores.

The multivariate models built for each domain of the child development measured at 2 years of age (Table 5) were statistically significant (*P* < 0·01) and had an adjusted *R* ^2^ value ranging between 5 and 7 % (adjusted *R* ^2^ values of cognition, expressive language and receptive language domains were 4·6, 7·2 and 4·7 %, respectively). Considering the collinearity between the body Fe and Hb (correlation coefficient, *r* = 0·63), the Hb was excluded from the final model. High mean BLL of 15 and 24 months was associated with lower cognition and expressive language raw scores at 2 years of age, which remained significant in the multivariate analysis with sex, mean body Fe (of 7, 15 and 24 months), maternal intelligence and SES (Table 5). Mean body Fe did not have any association with either cognition, expressive or receptive language scores at 2 years of age. Other factors associated with the 2 year development in the multivariate analysis were the SES associated with both cognition and expressive language; girls had better expressive language than boys, and higher maternal intelligence was associated with better receptive language.

**Table 5:** Factors associated with cognition, expressive and receptive language components of Bayley Scales of Infant and Toddler Development III (BSID III) raw scores assessed at 2 years (*N* 226*)

	Unadjusted beta coefficient	95 % CI	*P* †	Adjusted beta coefficient	95 % CI	*P* †
Cognition						
Sex	–0·1	–0·9, 0·9	0·977	–0·3	–1·2, 0·6	0·529
Mean body Fe‡	0·1	–0·1, 0·2	0·131	0·1	–0·1, 0·2	0·306
Mean blood Pb§	–0·1	–0·2, –0·02	**0·009**	–0·2	–0·2, –0·03	**0·009**
Mother’s IQ scores	0·1	–0·01, 0·06	0·203	0·01	–0·03, 0·05	0·712
WAMI	1·5	0·5, 2·4	**0·002**	1·4	0·4, 2·4	**0·006**
Expressive language						
Sex	1·7	0·5, 2·3	**0·006**	1·4	0·2, 2·7	**0·020**
Mean body Fe‡	0·1	–0·02, 0·3	0·111	0·04	–0·1– 0·2	0·548
Mean blood Pb§	–0·2	–0·3, –0·06	**0·003**	–0·2	–0·3, –0·1	**0·006**
Mother’s IQ scores	0·04	–0·02, 0·09	0·64	0·1	–0·5, 0·07	0·673
WAMI	1·6	0·3, 2·9	**0·015**	1·5	0·09, 2·8	**0·036**
Receptive language						
Sex	0·4	–0·2, 1·1	0·188	0·2	–0·4, 0·9	0·473
Mean body Fe‡	0·07	–0·02, 0·2	0·125	0·03	0·05, 0·1	0·408
Mean blood Pb§	–0·04	–0·1, 0·02	0·185	–0·04	–0·1, 0·02	0·205
Mother’s IQ scores	0·05	0·02, 0·08	**0·001**	0·04	0·01, 0·07	**0·007**
WAMI	0·8	0·6, 1·5	**0·034**	0·4	–0·3, 1·1	0·294

IQ, intelligence quotient; WAMI, Water and sanitation, Assets, Maternal education and Income score for socio-economic status.

* Of the 228 children who completed 2 years of follow-up, two subjects had missing data either in blood Pb levels or in development scores; hence, the linear model includes data for only 226 children.

† Significant associations in unadjusted and adjusted analyses are presented in bold letters.

‡ Mean cumulative body Fe of 7, 15 and 24 months.

§ Mean cumulative blood Pb levels of 15 and 24 months.

Linear models constructed for each domain of cognition assessed at 5 years of age (Table 6) were statistically significant (*P* < 0·0001) and had an adjusted *R* ^2^ value ranging between 10 and 13 % (adjusted *R* ^2^ values of verbal, performance and processing speed domains were 9·8, 12·3 and 12·7 %, respectively). Body Fe status of early childhood (cumulative values of 7, 15 and 24 months) had a significant association with verbal, performance and processing speed at 5 years of age, which remained significant in the multivariate analysis (Table 6). Mean BLL did not have a significant association with any of the domains of cognition assessed at 5 years of age. Other factors associated with the 5 year cognition in the multivariate analysis were the maternal intelligence affecting verbal, performance and processing speed components and SES the performance domain.

**Table 6:** Factors associated with verbal, performance and processing speed components of Wechsler Preschool Primary Scales of Intelligence IV (WPPSI IV) raw scores assessed at 5 years (*N* 212)

	Unadjusted beta coefficient	95 % CI	*P* *	Adjusted beta coefficient	95 % CI	*P* *
Verbal						
Sex	1·1	–1·4, 3·6	0·379	–0·1	–2·5, 2·4	0·978
Mean body Fe†	0·5	0·2, 0·9	**0·001**	0·4	0·1, 0·7	**0·011**
Mean blood Pb‡	–0·1	–0·3, 0·1	0·423	–0·1	–0·3, 0·1	0·462
Mother’s IQ scores	0·3	0·1, 0·4	<**0·0001**	0·2	0·1, 0·3	<**0·0001**
WAMI category	3·1	0·4, 5·7	0·025	0·9	–1·9, 3·6	0·524
Performance						
Sex	–0·3	–2·9, 2·3	0·813	–1·73	–4·2, 0·8	0·182
Mean blood Fe†	0·6	0·3, 0·96	**<0·0001**	0·5	0·2, 0·8	**0·003**
Mean blood Pb‡	–0·2	–0·5, 0·02	0·071	–0·2	–0·5, –0·1	**0·056**
Mother’s IQ scores	0·2	0·1, 0·3	**<0·0001**	0·2	0·04, 0·3	**0·009**
WAMI category	4·9	2·1, 7·8	**<0·0001**	3·1	0·2, 5·9	**0·037**
Processing speed						
Sex	6·5	1·6, 11·4	**0·010**	4·3	–0·4, 9·1	0·076
Mean body Fe†	1·3	0·6, 1·9	**<0·0001**	0·9	0·2, 1·5	**0·007**
Mean blood Pb‡	–0·16	–0·7, 0·4	0·493	–0·1	–0·5, 0·3	0·682
Mother’s IQ scores	0·5	0·2, 0·7	**<0·0001**	0·3	0·1, 0·6	**0·005**
WAMI category	8·7	3·3, 13·9	**0·002**	4·7	–0·7, 10	0·090

IQ, intelligence quotient; WAMI, Water and sanitation, Assets, Maternal education and Income score for socio-economic status.

* Significant associations in unadjusted and adjusted analyses are presented in bold letters.

† Mean cumulative body Fe of 7, 15 and 24 months.

‡ Mean cumulative Pb levels of 15, 24 and 36 months.

## Discussion

This prospective birth cohort follow-up study from urban Vellore provided the trends of Hb, Fe and Pb levels during the early childhood. Further, a negative association of high BLL with cognition and expressive language domains of child development was demonstrated. The current study also showed a negative association of ID with verbal, performance and processing domains of cognition. Retention in the current study was good at all planned time points. There was minimal drop out and 212 children presented at 5 years of age (Table 1). Our finding of high prevalence of anaemia in more than half of the study children at both 7 and 15 months of age has been reported from other studies and reviews^(23–26)^. The proportion of anaemia attributable to ID was only 18·7 % at 7 months. ID anaemia became the commonest cause of anaemia at 15 months of age and remained so at 24 months, despite a drop in overall prevalence of anaemia and improvement in Fe stores in the later age group. Prevalence of anaemia in Indian children aged 6–59 months was estimated to be 58·5 and 69·4 % in National Family Health Survey (NFHS) conducted during 2015–2016 (NFHS-4) and 2005–2006 (NFHS-3), respectively. Although the prevalence of anaemia had lowered to 58·5 % in NFHS-4, from its previous estimate of 69·4 % in NFHS-3, anaemia continues to be a public health problem in India^(27)^. Other studies from India also had reported that anaemia was common in the older age group with a prevalence ranging between 27 and 63 %^(28,29)^. Other causes of anaemia in children from low–middle income countries are hookworm infestation with resultant blood loss, vitamin B_12_ deficiency manifesting as megaloblastic anaemia, haemoglobinopathy disorders and environmental enteropathy^(23,30,31)^. However, the association between above-mentioned causes of anaemia and cognitive development was not investigated in the current analysis.

Young children showed the lowest body Fe status at 15 months of age probably due to a combination of factors such as increased Fe demand to support the expanding blood volume requirements, depletion of antenatal Fe stores and sub-optimal Fe intake^(23)^.The estimated daily requirement of absorbed Fe in the first year of life ranges between 0·55 and 0·75 mg and cannot be fully met by breast milk, which has a mean Fe level of 0·4 mg/l^(32)^. Typical weaning foods used in South India are rice- and milk-based and contain low Fe. Introduction of animal source foods, which can be a good source of dietary Fe, into a child’s diet is usually later in this community, unlike other communities where meat is added earlier on^(26)^. This might explain the lowest body Fe status shown at 15 months of age and is corroborated with other low–middle income countries studies^(32,33)^. Fe status improved in the second year in this study, probably due to the child eating more variety of food from the family pot with a decrease in consumption of milk and milk products^(26)^.

Most of the Fe in our body is bound in haem proteins and storage proteins, and around 3 % is bound with critical enzyme systems such as cytochromes and peroxidases. ID manifesting during the early childhood, when there is biologically an increased demand for Fe, can be detrimental especially to critical Fe-dependent enzymes^(34)^. It is possible therefore that ID can cause deficiencies in mitochondrial function, neurotransmitter synthesis and neuronal maturation in the brain, which can have long-lasting effects on cognition^(4,35–37)^.

High BLL as in the current study have been reported from India^(38–40)^. Previous studies had used BLL of 10 µg/dl (0·48 µmol/l) as the cut-off for high level; but the revised cut-off of 5 µg/dl (0·24 µmol/l) has been used for the current analysis^(6,7,41)^. This current community-based prospective study shows increasing Pb levels from 15 to 36 months of age, with high Pb levels present in 97 % of children (>5 µg/dl (0·24 µmol/l)) at 36 months of age. This has been reported also from Nepal, in a hospital-based study of 6–36 months old children, where 64·4 % had high BLL of >5 µg/dl (0·24 µmol/l)^(42)^. Risk factors associated with high BLL include low SES, industrial area exposure, playing with dirt and mud, exposure to enamel paints, piped water supply and mud/clay floors of the house; the last two factors being shown as predictors for high BLL in this birth cohort in an evaluation published earlier^(10,38–44)^. There is evidence suggesting transplacental transfer of Pb, with sixteen (84·2 %) cord blood samples obtained from nineteen mothers, who had high BLL during their antenatal period, had elevated Pb levels^(45)^. This highlights the possibility of Pb exposure even as early as the *in utero* phase of life. In the current study, the continued environmental exposure to Pb during early childhood could have caused increasing BLL trends.

There can be interactions between Pb and Fe. ID can cause pica, which can exaggerate Pb exposure through mouthing of inanimate objects^(8,9)^. Heavy metals including Pb share intestinal Fe absorption pathways, where there can be competitive inhibition between Pb and Fe^(8)^. The classic clinical manifestation of Pb poisoning in children is anaemia, more specifically hypochromic microcytic anaemia^(41)^. The prevalence and severity of anaemia correlate directly with BLL. Younger and Fe-deficient children are at higher risk of developing Pb-induced anaemia. Pb toxicity results in anaemia through two mechanisms – by causing direct damage to the cell membrane of erythrocytes resulting in haemolysis and through inhibition of the enzyme delta-aminolevulinic acid dehydratase in the Fe-dependent haem synthesis pathway^(41,46)^. Inhibition of delta-aminolevulinic acid dehydratase enzyme not only results in anaemia by affecting haem synthesis but also results in the accumulation of haem precursors such as aminolevulinic acid, which can cause harmful effects to the nervous system^(46,47)^.

In the current study, high mean BLL over 15 and 24 months had negative association with both cognition and expressive language raw scores of 24 months. Literature shows that early childhood Pb exposure can adversely affect later age cognitive capability – a doubling of BLL or tooth Pb levels at 2 years of age can cause a mean deficit of 1–2 intelligence quotient points at 5 years of age^(48,49)^. Pb even in smaller dose exposure is toxic to the nervous system and can impair cognition and behaviour; thus the recommendation that there is no safe BLL^(6,41)^. Pb interferes with ion channels in the brain by its function as a Ca analogue^(6,46)^. In addition, Pb increases oxidative stress by generation of reactive oxygen species and inactivates antioxidants such as glutathione by binding to them^(50,51)^. Pb can accumulate over time as shown in the current study and can have a negative association with cognition and learning such as impaired visual-motor integration, longer reaction time and reading disability in school age years; which persist into young adulthood^(40,49)^.

The finding of the current study is that early childhood cumulative body Fe status had no association with development at 2 years of age, but was associated with verbal, performance and processing speed components of cognition at 5 years. This may be due to the fact that cognition is a stable construct at 5 years as frontal lobes are better myelinated and mature at this age^(1,52)^. Early onset ID through its impact on the activity of Fe containing enzymes can affect neuronal myelination, mitochondrial function and neurotransmitter metabolism of developing brain, which can have long-term effects on cognition and behaviour of children^(3,4,34,53)^. Subsequent Fe supplementation beyond the early childhood can optimise body Fe stores, but cannot neutralise the impact of early onset ID on neurodevelopment, cognition and behaviour^(2,53,54)^. ID in school age years can also affect cognition, as per other Indian studies^(24,28,30,55)^. Among cognitive functions, ID impairs executive function the most, which includes but not limited to attention, inhibitory control, planning ability and working memory^(3,4,36,54)^. ID can also cause concentration and attention problems, tiredness and low mood in school-age years; which can improve on appropriate Fe supplementation^(3,54)^. ID without anaemia in women of reproductive age group was shown to affect their executive function, indicating the need to evaluate ID on its own without the ramification of presence or absence of anaemia^(56)^. Antenatal or early childhood Fe supplementation shows inconsistent effects on both early- and long-term cognition in children^(37,57,58)^.

Other factors that have association with development and cognition in the current study are maternal cognition, SES factors and sex. Maternal intelligence can affect child cognition not just by genetic influence^(59)^ but also by providing optimal macro- and micro-environments including appropriate play materials and stimulating experiences^(15,60,61)^. Improvement in SES factors such as access to safe water and sanitation, better maternal education status and improvement of family economy can offer better experiences for children to grow and develop^(17)^. Repeated diarrhoeal illnesses predisposed by lack of safe water and sanitation facilities in the early childhood can have direct negative impact on child development and cognition. Indirect impact of diarrhoeal illnesses on cognition is through manifestation of nutritional deficiencies following these illnesses, which in turn affects cognitive development. In addition to these infections, exposure of toxins such as Pb can further affect the cognition^(15,61)^. Girls tend to have better speech and expressive language skills than boys at 2 years of age, as shown in the current analysis, probably due to better environmental interactions^(62,63)^.

The current study has strengths such as longitudinal birth cohort follow-up, standardised developmental and cognitive assessments by a single trained psychologist, rigorous quality control with internal and external reviews, standardised laboratory assessments and adjustment for SES. However, regression models could explain only up to 13 % of the variance associated with the predicted outcomes, highlighting the presence of other factors that could have influenced the outcome. A comparatively smaller sample size prevented a detailed analysis of other factors within the model. Inclusion of factors such as years of preschool education could have made the model more explanatory. Due to the collinearity between serum Fe and Hb levels, the latter was not included in the regression; which can also be a limitation to the current study. Despite these limitations, this prospective birth cohort study showed that high BLL in early childhood was negatively associated with development at 2 years of age and the association of early childhood ID with cognition at the age of 5 years. Future studies can explore the contribution of other early childhood micronutrient deficiency and environmental toxicity to development and cognition, and the ideal timing of nutritional interventions such as Fe supplementation and fortification.

## Significance

This longitudinal birth cohort shows maximum ID at 15 months of age and increasing BLL with age in a low–middle income country setting.

Early childhood ID shows a negative association with verbal, performance and processing speed domains of cognition at 5 years of age, while high BLL was negatively associated with cognition and expressive language at 2 years of age.
